# Perforative peritonitis confused with peritoneal dialysis-related peritonitis: Report of three cases

**DOI:** 10.1016/j.ijscr.2020.03.046

**Published:** 2020-04-22

**Authors:** Ryosuke Arata, Masataka Banshodani, Masahiro Yamashita, Sadanori Shintaku, Misaki Moriishi, Hideki Kawanishi

**Affiliations:** Department of Artificial Organs, Akane-Foundation, Tsuchiya General Hospital, Japan

**Keywords:** PD, peritoneal dialysis, HD, hemodialysis, CT, computed tomography, CRP, C-reactive protein, WBC, white blood cell, Case series, Gastrointestinal perforation, Peritoneal dialysis, Refractory peritonitis

## Abstract

**Introduction:**

Perforative peritonitis in patients on peritoneal dialysis (PD) is a serious adverse event associated with significant mortality. The signs and symptoms of perforative peritonitis in patients on PD are often confused with those of PD-related peritonitis; therefore, early diagnosis is often difficult.

**Presentation of cases:**

In all three cases, antibiotic therapy was started for peritonitis. Although contrast-enhanced computed tomography (CT) was not performed, perforative peritonitis was suspected due to severe cloudiness of PD effluents, and emergency surgeries were performed 8, 5, and 6 days after therapy onset in cases 1, 2, and 3, respectively. In case 1, the ileum was perforated owing to ischemia, and partial ileal resection and divided ileostomy were performed. The patient died 18 days postoperatively. In case 2, partial ileal resection and divided ileostomy were performed for an incarcerated obturator hernia and perforated ileum. The patient was transferred for hemodialysis (HD) and discharged 117 days postoperatively. In case 3, lavage drainage was performed for peritonitis because of mesenteric penetration of a sigmoid colon diverticulum. The patient was then transferred for HD, and colostomy was subsequently performed. He was discharged 159 days postoperatively.

**Discussion:**

Early diagnosis between PD-related peritonitis and perforative peritonitis is often difficult since the washing effect of the peritoneal dialysate might relieve peritoneal irritation.

**Conclusion:**

In PD patients with refractory peritonitis, it is necessary to keep in mind the possibility of perforative peritonitis, and the differential diagnosis should be performed using contrast-enhanced CT within at least 5 days after antibiotic therapy.

## Highlights

• Intestinal perforation in patients on peritonitis dialysis (PD) has high mortality.
• Perforative peritonitis in PD patients and PD-associated peritonitis patients have similar signs.
• Rapid diagnosis can help exclude perforation in cases of refractory peritonitis.

## 1 Introduction

Peritonitis remains a major complication, leading to possible membrane damage and even death [1]. Although touch contamination during peritoneal dialysis (PD) effluent exchange is the most common cause of PD-related peritonitis [2], perforative peritonitis is also a potentially fatal complication. Despite the need for appropriate surgical intervention, early diagnosis of perforative peritonitis is difficult because the washing effect of the peritoneal dialysate might relieve peritoneal irritation [3]. In PD patients with refractory peritonitis, early diagnosis is important because of the possibility of perforative peritonitis.

This work has been reported in line with the PROCESS criteria [4]. Research registry of the case series: #researchregistry5417.

## 2 Case presentation

### 2.1 Case 1

**Fig. 1:**
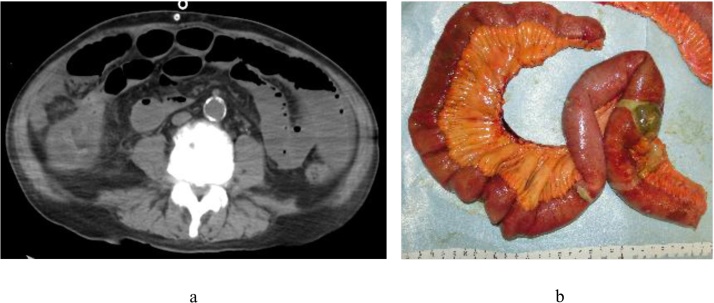
(a) Computed tomography (CT) findings. CT reveals intestinal dilatation, but no evidence of free air or gastrointestinal perforation. (b) The excised specimen showing ischemic changes and a perforation in the ileum.

A 67-year-old man began PD for diabetic nephropathy at age 63. He had suffered three episodes of PD-related peritonitis, all of which improved with antibiotic therapy. During the course of an unrelated hospitalization, he presented with cloudiness of the PD effluent. His symptom was only mild abdominal distention, however, serum C-reactive protein (CRP) level and white blood cell (WBC) count in the PD effluent were 15.91 mg/dL and 4,688 cells/μL (neutrophils, 78.7%), respectively. He was diagnosed with peritonitis and was started on intravenous tazobactam/piperacillin and intraperitoneal tobramycin for broad-spectrum coverage (day 0). WBC count in the PD effluent initially decreased to 2,407 cells/μL (day 1), but subsequently increased to 3,064 cells/μL, and the antibiotic was changed to levofloxacin (day 2). Enterococcus growth was observed on culture tests, and the antibiotic was changed to vancomycin (day 3). He complained of abdominal pain, and non-contrast computed tomography (CT) revealed intestinal dilatation but no free air in the abdominal cavity (Fig. 1a) (day 7). Ischemic enteritis was suspected due to hematochezia, and emergency surgery was performed due to the exacerbation of abdominal pain and severe cloudiness of the PD effluent (day 8). On inspection of the abdominal cavity, ischemic changes and partial perforation were observed in the ileum; he was diagnosed with perforative peritonitis associated with ischemic enteritis (Fig. 1b). He underwent partial ileal resection and divided ileostomy, and was transferred to hemodialysis (HD). However, his circulatory dynamics were unstable and he died of arrhythmia 18 days after the surgery.

### 2.2 Case 2

**Fig. 2:**
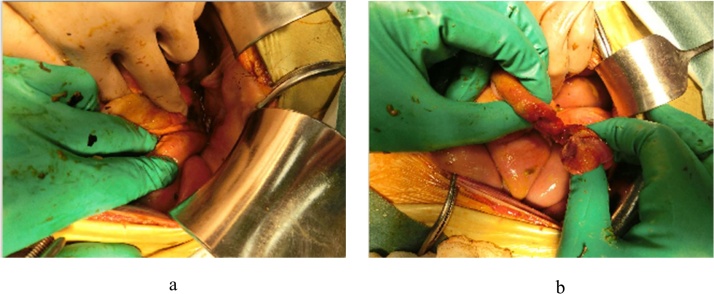
Intraoperative findings. (a) An incarcerated obturator hernia was found in the ileum. (b) The ileum was found to be torn once the incarceration was released.

A 72-year-old woman began PD for diabetic nephropathy at age 71. She had no earlier episodes of PD-related peritonitis. She was hospitalized for right hip pain and was suspected of having sciatica; thus, rehabilitation was begun. During the course of hospitalization, she presented with cloudiness of the PD effluent but no abdominal pain. Serum CRP level and WBC count in the PD effluent were 0.79 mg/dL and 179 cells/μL (neutrophils, 74.3%), respectively. She was diagnosed with peritonitis and began receiving intravenous tazobactam/piperacillin and intraperitoneal tobramycin for broad-spectrum coverage (day 0). WBC count in the PD effluent increased to 1,395 cells/μL (day 2). The antibiotic was changed to vancomycin after Gram-positive cocci were found in culture tests (day 3). Although contrast-enhanced CT was not performed, perforative peritonitis was suspected due to the occurrence of abdominal pain and severe cloudiness of the PD effluents, and emergency surgery was performed (day 5). Inspection of the abdominal cavity revealed an incarcerated obturator hernia in the ileum (Fig. 2a). The ileum was torn upon release of the incarceration (Fig. 2b). She underwent partial resection of the ileum, divided ileostomy, and the creation of a mucus fistula. The PD effluent culture was negative for bacteria by day 6. The patient was transferred to HD and later discharged 117 days after the surgery.

### 2.3 Case 3

**Fig. 3:**
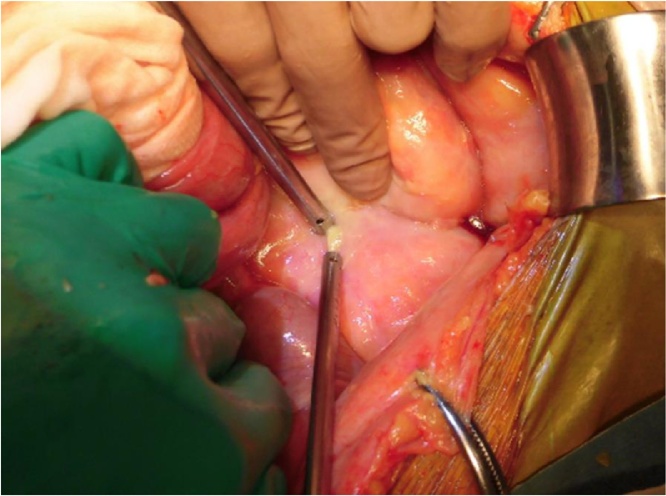
Intraoperative findings. A mesenteric abscess was found beside a perforated sigmoid diverticulum.

A 56-year-old man started PD for nephrosclerosis at age 42. He had no earlier episodes of PD-related peritonitis. Although he had no abdominal pain, he presented with cloudiness of the PD effluent. At admission, serum CRP level and WBC count in the PD effluent were 27.81 mg/dL and 1,238 cells/μL (neutrophils, 92.0%), respectively. He was diagnosed with peritonitis and began receiving intravenous tazobactam/piperacillin and intraperitoneal tobramycin for broad-spectrum coverage (day 0). WBC count in the PD effluent initially decreased to 432 cells/μL (day 1), but subsequently increased to 623 cells/μL (day 2). Because of the severe cloudiness of the PD effluent, this treatment was changed to vancomycin (day 5). Although contrast-enhanced CT was not performed, emergency surgery was performed due to the occurrence of abdominal pain and rapid increase of WBC count in the PD effluent (45,019 cells/μL) (day 6). At laparotomy, a mesenteric abscess was found beside a perforated sigmoid diverticulum. He underwent intraperitoneal drainage (Fig. 3). The PD effluent culture was negative for bacteria (day 9). After the surgery, the patient was transferred to HD and later underwent a colostomy at the level of the transverse colon. He was discharged 159 days after the construction of the colostomy.

## 3 Discussion

Peritonitis in PD patients is predominantly PD-related peritonitis, but perforative peritonitis in PD patients is a severe complication with a mortality rate of 46.3% [5]. Therefore, it is important to obtain a differential diagnosis between PD-related peritonitis and perforative peritonitis. However, the initial symptoms of perforative peritonitis are often confused with those of PD-related peritonitis since the washing effect of the peritoneal dialysate might relieve peritoneal irritation [3]. In our cases, at the time of peritonitis diagnosis, there was mild abdominal distention in only case 1. In cases 2 and 3, there were no abdominal symptoms until just before surgery. In case 2, right hip pain may have been a symptom of obturator hernia. In perforative peritonitis in PD patients, it may be difficult to perform a differential diagnosis based on only abdominal symptoms, such as peritoneal irritation.

In most cases, PD-related peritonitis usually improves within 72 h after the initiation of antibiotic therapy [6]. A retrospective study showed that WBC count in the PD effluent ≥1,090 cells/μL 2 days after the start of antibiotic therapy was an independent prognostic marker for treatment failure [7]. In our cases, WBC counts in the PD effluent 2 days after the start of antibiotic therapy were 3,064, 1,395, and 623 cells/μL in cases 1, 2, and 3, respectively. These findings suggest that it may be impossible to perform a differential diagnosis based on WBC count in the PD effluent. The International Society for Peritoneal Dialysis (ISPD) guidelines define “refractory peritonitis” as a situation where the contamination of the PD effluent does not disappear 5 days after the use of appropriate antibiotics; they recommend that in this situation, the PD catheter should be promptly removed [6]. In our cases, on days 8, 5, and 6 from the start of therapy in cases 1, 2, and 3, respectively, surgeries were performed. Therefore, perforated peritonitis should be suspected within at least 5 days after the initiation of antibiotic therapy in PD patients with refractory peritonitis.

It may be desirable to perform contrast-enhanced CT to rule out perforative peritonitis. Hainaux et al. suggested that three CT findings have a high predictive value for the site of perforation: concentrated bubbles of extraluminal air in close proximity to the bowel wall; a focal defect in the bowel wall; and segmental bowel wall thickening [8]. In another report, intraperitoneal free air, a sign of perforative peritonitis, was revealed on CT in 30% of PD patients without perforative peritonitis [9]. Moreover, Fujii et al. suggested that CT cannot be used as a diagnostic tool for the early identification of perforation peritonitis in PD patients [10]. However, contrast-enhanced CT should be used because it also provides information other than the presence of free air. At the time of the CT scan in case 1, perforative peritonitis might not have occurred. If contrast-enhanced CT had been performed, ischemic enteritis would have been evident; thus, other therapy such as interventional radiology might have been possible. In addition, the CT scan has superior sensitivity and accuracy compared to other radiological examinations to assess the presence of an obturator hernia [11]. For a diverticulum, a CT scan is the most accurate and recommended examination for diagnosis [12]. If CT scans had been taken at the diagnosis of refractory peritonitis in cases 2 and 3, surgical interventions could have been applied earlier and better results might have been obtained. Therefore, in PD patients with refractory peritonitis, the differential diagnosis should be rapidly performed using contrast-enhanced CT.

Several other methods for performing a differential diagnosis between PD-related peritonitis and perforated peritonitis have been reported. ISPD recommendations on peritonitis suggest that surgical evaluation should be obtained immediately if multiple enteric organisms (multiple gram-negative or mixed gram-negative/gram-positive organisms) are observed to grow in culture tests of PD effluents. There are also reports that when *Klebsiella oxytoca* is detected in PD effluent, the digestive tract should be examined for infection [13]. Some reports suggest that amylase levels in PD effluent are useful for determining perforative peritonitis [14,15].

## 4 Conclusion

In PD patients with refractory peritonitis, it is necessary to keep in mind the possibility of perforative peritonitis. Therefore, in these cases, the differential diagnosis should be performed using contrast-enhanced CT within at least 5 days after antibiotic therapy.

## Declaration of Competing Interest

The authors have no conflicts of interest.

## Funding

This research did not receive any specific grant from funding agencies in the public, commercial, or not-for-profit sectors.

## Ethical approval

Ethical approval was not required and patient identifying knowledge was not presented in the report.

## Consent

Written informed consent has been obtained from the patient for the publication of this case report and any accompanying images.

## Author contribution

RA and MB participated in treatment of the patient, collected case details, literature search and draft the manuscript. MY and SS participated in treatment planning of the patient. HK participated in treatment planning of the patient and helped to draft the manuscript. All authors read and approved the final manuscript.

## Registration of research studies

Research registry of the case series: #researchregistry5417.

## Guarantor

Masataka Banshodani.

## Provenance and peer review

Not commissioned, externally peer-reviewed.

## CRediT authorship contribution statement

**Ryosuke Arata:** Conceptualization, Methodology, Software, Validation, Formal analysis, Investigation, Resources, Data curation, Writing - original draft, Writing - review & editing, Visualization. **Masataka Banshodani:** Conceptualization, Methodology, Validation, Data curation, Writing - original draft, Writing - review & editing, Supervision, Project administration. **Masahiro Yamashita:** Validation. **Sadanori Shintaku:** Validation. **Misaki Moriishi:** Validation. **Hideki Kawanishi:** Validation, Writing - review & editing, Conceptualization.
